# Ketamine in clinical practice: transitioning from anesthetic agent to psychiatric therapeutic

**DOI:** 10.1017/S1092852925100333

**Published:** 2025-06-30

**Authors:** Jose Manuel Quintero, Rosa Helena Bustos, Sharon Lechtig-Wassermann, Stephania Beltran, Carlos A. Zarate

**Affiliations:** 1 Department of Clinical Pharmacology, Evidence-Based Therapeutics Group, Faculty of Medicine, Universidad de La Sabana and Clínica Universidad de La Sabana, Chía, Colombia; 2 Doctoral Programme of Biosciences, Universidad de La Sabana, Chía, Colombia; 3 Faculty of Medicine, Universidad de La Sabana, Chía, Colombia; 4 Experimental Therapeutics and Pathophysiology Branch, National Institute of Mental Health, National Institutes of Health, Bethesda, MD, USA

**Keywords:** anesthetic, antidepressant, recreational drug

## Abstract

Ketamine, originally synthesized in 1962, has gained increasing attention due to its rapid and sustained antidepressant effects in treatment-resistant depression (TRD). Unlike traditional antidepressants, ketamine acts on multiple neurotransmitter systems, including NMDA receptor antagonism and AMPA receptor potentiation, offering a novel mechanism for mood regulation. Beyond psychiatry, ketamine remains a valuable anesthetic and analgesic agent with applications in acute and chronic pain management. Its anti-inflammatory, neuroprotective, and potential antitumor properties further underscore its versatility in clinical medicine. Despite its therapeutic promise, ketamine poses challenges due to its dissociative and hallucinogenic effects, contributing to widespread recreational use. Chronic misuse is associated with adverse health outcomes, including bladder dysfunction and hepatobiliary complications. As a result, ketamine has been subject to increasing regulatory scrutiny, balancing its medical utility with concerns about abuse potential. The rediscovery of ketamine’s antidepressant effects in the early 2000s has revolutionized psychiatric treatment, particularly in TRD and suicidality. Recent studies have also explored its efficacy in bipolar depression, posttraumatic stress disorder, obsessive-compulsive disorder, and substance use disorders, expanding its therapeutic applications. Additionally, ongoing research aims to elucidate its long-term effects and optimize dosing regimens to maximize clinical benefits while minimizing risks. This review highlights ketamine’s multifaceted pharmacological properties, its evolution from an anesthetic to a novel psychiatric treatment, and its broader medical applications. As research advances, a comprehensive understanding of ketamine’s mechanisms and clinical implications will be crucial for optimizing its therapeutic potential while mitigating its risks.

## Introduction

Ketamine, originally synthesized in 1962, has recently gained significant attention owing to its unique pharmacological properties, particularly its rapid and long-lasting antidepressant effects in patients with treatment-resistant depression (TRD).^1^ ^–^^3^ Its ability to act on multiple neurotransmitter systems distinguishes it from traditional antidepressants, offering new hope to patients who do not respond to conventional therapies.^4^ Beyond its role in mental health, ketamine continues to be valued in medical settings for its anesthetic and analgesic properties.^5^

Despite these promising therapeutic applications, ketamine presents several challenges. Its dissociative and hallucinogenic effects have contributed to its popularity as a recreational drug, particularly among young people in party and club environments.^6^ Prolonged misuse can lead to adverse health outcomes such as bladder dysfunction and cholangiopathy.^7^ The rise in recreational ketamine use since the 1990s has prompted stricter regulations in many countries owing to concerns about abuse potential and the emergence of new analogs with unknown risks.^8^

Nevertheless, ketamine’s transformative impact on the treatment of depression and its established role in anesthesia highlight its dual potential as both a critical medical tool and a substance requiring careful regulation. Ongoing research and monitoring are essential to maximize its therapeutic benefits but minimize the risks associated with misuse. In this text, the term ketamine refers to its racemic form (*R,S*-ketamine). References to individual enantiomers are specified as *R*-ketamine (arketamine) or *S*-ketamine (esketamine).

### Discovery and development

In the mid-20th century, the quest for safer and more effective anesthetics spurred scientific exploration, culminating in the discovery of phencyclidine (PCP) through the Nobel Prize-winning Grignard reaction. Despite its initial promise as a safe anesthetic, PCP frequently induced prolonged delirium and sensory deprivation following surgical recovery.^9^ A significant breakthrough emerged in 1962 when Calvin Stevens of the Parke Davis laboratory synthesized Cl-581, later recognized as ketamine, from PCP.^10^ Ketamine was derived from PCP with the aim of lessening the serious psychotomimetic/psychodysleptic side effects and abuse potential of the parent drug, which was subsequently removed from the market in 1978.^11^ Its development was a pivotal moment that marked the inception of ketamine’s journey into medical science. Ketamine was first patented in Belgium in 1963 as a veterinary anesthetic. As early as 1964,^12^ Dr. Edward Domino and Dr. Guenter Corssen had initiated the first clinical investigations of ketamine, outlining its distinctive properties and effects in 20 male prisoners. Their observations revealed that subjects exhibited wakefulness with intact reflexes but remained unresponsive to sensory stimuli. After being patented by Parke-Davis for human and animal use in 1966, ketamine became available by prescription in 1969 as ketamine hydrochloride, under the name Ketalar. Ketamine was approved by the U.S. Food and Drug Administration (FDA) as a dissociative, rapid-acting, IV anesthetic in 1970^13^ and has been used in both human and veterinary medicine since then. Its approval was based on its rapid onset of action, favorable safety profile, and minimal respiratory depression compared with other anesthetic agents. Initially, it was approved solely as an anesthetic agent for diagnostic and surgical procedures.^12^ Although the World Health Organization (WHO) placed ketamine on its list of essential medications in 1985—where it has remained ever since—its use as a general anesthetic began to decline in clinical practice in the 1980s, mainly due to its psychotropic effects, such as hallucinations, dissociation, and emergence delirium during anesthetic recovery.^14^

Despite its waning popularity in medical circles, ketamine’s journey took an unexpected turn in the 1970s with the emergence of reports suggesting a “psychedelic effect” associated with subanesthetic doses.^15^ This revelation catalyzed its transition into a recreational substance, marking the genesis of its association with the burgeoning rave culture of the 1980s.^12^ Ketamine swiftly assumed various street names and became a staple “club drug,” leading to its classification as a Schedule III substance.^10^ The early 1980s witnessed the advent of the “emergence phenomenon,” characterized by heightened illicit usage and a subsequent departure from mainstream medical applications.^12^ However, the 1990s heralded a resurgence in ketamine’s medical utility, fueled by a deeper comprehension of its mechanism of action and therapeutic efficacy.^10^

The turn of the millennium ushered in a new era for ketamine, marked by the pioneering work of Zarate *et al.*, which were the first to use ketamine as an antidepressant in patients with TRD.^16^ Concurrently, continuous ketamine infusions gained traction as a management strategy for complex regional pain syndrome (CRPS) thanks to the groundbreaking efforts of various research groups.^17^ ^,^^18^ However, this period was not without regulatory interventions, as evidenced by ketamine’s reclassification from Schedule H to Schedule X in 2013 under the Drugs and Cosmetics Act. This legislative maneuver sought to curb its misuse and restrict access.^12^

### Contributions to medicine

Ketamine remains indispensable in anesthesia, with established roles as a sedative, amnestic, and analgesic agent. Strong evidence supports its efficacy in managing traumatic brain injury,^19^ acute pain,^20^ and chronic pain.^21^ Ketamine’s clinical applications have since expanded to encompass its neuroprotective,^22^ ^,^^23^ anti-inflammatory^24^ ^,^^25^ and antitumor properties.^26^ ^,^^27^ However, limited data exist regarding its use in conditions such as elevated intraocular pressure, cancer pain, and critical care settings, highlighting the need for further research to fully elucidate its clinical utility.^28^

The seminal work of Skolnick and Trullas (1990),^29^ who first suggested that the glutamatergic system might play a role in depression, laid the foundation for subsequent research in this area. One early study of 29 patients with treatment-resistant schizophrenia found that ketamine improved depressive inhibition and apathetic-abulic states without significant side effects.^30^ Lv et al. later demonstrated that ketamine induces persistent reconfiguration of brain networks, notably by downregulating connectivity in reward circuits, counteracting depressive alterations, and highlighting targets for circuit-specific therapeutics.^31^ This evolution exemplifies the dynamic interplay between scientific discovery, clinical applications, and societal acceptance.

In recent years, research regarding the multifaceted therapeutic potential of ketamine has increased. As noted above, Zarate *et al.* pioneered its use in TRD, where it demonstrated rapid and robust antidepressant effects.^16^ Specifically, ketamine was found to exert its antidepressant effects within hours compared to weeks to months typically required by traditional antidepressants such as selective serotonin reuptake inhibitors and tricyclic antidepressants. These findings were later extended to bipolar depression by Diazgranados *et al.* ^32^ and Zarate *et al.* ^33^ Further studies from Lally *et al.* highlighted ketamine’s impact on anhedonia,^34^ suggesting broader applications beyond mood disorders.^35^ In parallel, Price *et al.* at Mount Sinai investigated ketamine’s efficacy in reducing suicidal ideation^36^; given that ketamine acts within a few hours rather than within weeks, its potential use as a rapid-acting antidepressant for patients at high suicidal risk has significant public health implications.^13^ Other studies have explored ketamine’s potential for treating posttraumatic stress disorder (PTSD),^37^, obsessive-compulsive disorder,^38^ cocaine and alcohol use disorders,^39^ and social anxiety disorders,^40^ reflecting its expanding therapeutic applications. Ongoing research is also currently exploring ketamine’s potential for sustained antidepressant effects.^41^ ^,^^42^

## Pharmacology of ketamine

Although ketamine primarily exerts its effects through non-competitive N-methyl-D-aspartate (NMDA) receptor antagonism, its mechanism of action extends beyond NMDA receptor antagonism,^43^ to include downstream effects on α-amino-3-hydroxy-5-methyl-4-isoxazolepropionic acid (AMPA) receptor activation^44^ brain-derived neurotrophic factor (BDNF) signaling,^45^ and synaptic plasticity,^46^ reflecting a complex neurobiological cascade.

### Chemistry and mechanism of action

Ketamine is a phenylcyclohexylamine derivative (mol. wt. = 237.73) consisting of two optical enantiomers, (*S*)- and (*R*)-ketamine.^24^ Used as a chlorhydrate in a slightly acid (pH 3.5–5.5) aqueous solution, ketamine sometimes includes benzethonium chloride or chlorobutanol as preservatives.^47^ The neuropharmacology of ketamine is complex and detailed below (Table 1).

**Table 1.** Chemical description and structural relationship activity of ketamine^48^ ^–^^50^

	Ketamine
Chemical structure	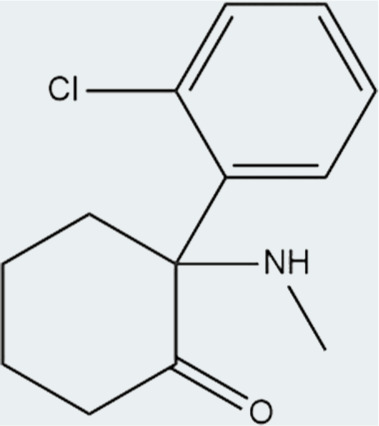
Chemical name	2-(2-chlorophenyl)–2-(methylamino)cyclohexan–1-one
Synonyms	Ketalar, Kalipsol, Ketamine Hydrochloride, Ketanest
Molecular formula	C_13_H_16_ClNO
Molecular weight	237.72 g/mol
Melting point	260 °C
Stereochemical description	Ketamine belongs to the arylcyclohexylamine class of compounds. The structure of ketamine contains a chiral center, with two optical isomers (enantiomers): (*S*)- and (*R*)-ketamine. These enantiomers exhibit different potencies and pharmacokinetics, with *R*-ketamine being less potent than *S*-ketamine. The cyclohexanone ring and the amino group at position 2 of the cyclohexanone ring are crucial for its anesthetic effect. Ketamine increases its solubility in its hydrochloride salt form.

### Pharmacokinetics

*Absorption:* Ketamine, which is both water- and lipid-soluble, can be administered through various routes, including oral, intranasal, sublingual, rectal, intramuscular, subcutaneous, intraosseous, intravenous, and inhaled. Oral administration results in significant first-pass metabolism, producing norketamine and dehydronorketamine. Rectal administration acts more quickly and is often used in children. Recreational use typically involves inhalation (insufflation).^51^

*Bioavailability:* Systemic clearance is 60–147 L/h/70 kg, which equals the liver blood flow, explaining the low bioavailability of oral ketamine (only 8–24%).^52^ By intravenous (I.V.) route, ketamine reaches its receptors very quickly with a transfer half‐life of less than 1 min. Intramuscular ketamine administration has high bioavailability (93%), with a plasma peak obtained in 5 min. However, its bioavailability is limited (20%) because of hepatic metabolism. The intrarectal and intranasal bioavailability of ketamine are ~25 and ~50%, respectively. The concentration peak occurs in 20–30 min after oral ingestion.^47^

*Distribution*: After systemic absorption, ketamine is rapidly distributed into the brain and other well-perfused tissues. Ketamine exhibits lipophilic properties that contribute to its pharmacokinetic profile. Pharmacokinetic analyses of ketamine revealed a biphasic elimination profile characterized by alpha and beta half-lives. Alpha half-life refers to the initial distribution phase, during which ketamine is rapidly redistributed from the bloodstream to highly perfused tissues, including the brain. In contrast, the beta half-life represents the elimination phase during which ketamine is metabolized and excreted from the body. These distinct phases help explain ketamine’s rapid onset of action and relatively short duration of effect following its intravenous administration. The alpha half-life ranges from approximately 5–17 min, while the beta half-life varies from around 180 min in healthy volunteers undergoing surgery to 300 min in critically ill patients. Distribution volume during the beta phase is about 5 L/kg in healthy surgical patients and increases to 16 L/kg in critically ill individuals. Clinically, this results in an immediate onset of action, with the peak analgesic effect (and associated elevation in blood pressure) occurring in less than 5 min. The duration of analgesia is typically under 5 min for doses ≤0.125 mg/kg, extending to 10–20 min with higher doses.^53^ A short alpha half-life and a short context-sensitive half-time are consistent with rapid recovery after intravenous ketamine anesthesia. It has a short alpha half-life (2–4 min) and a longer beta half-life (2–4 h) in humans. Plasma protein binding of ketamine is low (10–30%).^52^

*Metabolism:*

- 10–30% binding to plasma proteins.
- High liposolubility, 5 times higher than thiopental; hence, its extensive distribution.
- Distribution volume: at steady state is around 200 l 5, or 2.3 l/kg
- Mostly metabolized to norketamine (80%), an active metabolite that is itself principally hydroxylized in 6‐hydroxy‐norketamine (15%), finally excreted in bile and urine after glucuronidation.

Ketamine’s primary metabolic pathway involves N-demethylation by CYP3A4, producing the active metabolite norketamine, which retains anesthetic and some psychoactive effects. This helps maintain therapeutic efficacy even at lower ketamine blood levels. Both ketamine and norketamine undergo hydroxylation at carbons 3–6 of the cyclohexanone ring, forming inactive free and glucuronidated hydroxylated derivatives that are more water-soluble and easily excreted in urine.^51^

The average steady-state plasma concentration necessary to achieve anesthesia with ketamine was reported to be 2200 ng/ml, or 9.3 *μ*M. Oral (500 mg) or intrarectal (8–15 mg/kg) administration of ketamine suffice to induce sedation and/or general anesthesia in humans. Moreover, waking from ketamine-induced anesthesia occurs at plasma concentrations ranging from 640 to 1100 ng/ml or 2.7–4.7 *μ*M.^54^

*Elimination*: The elimination half-life is between 2 and 4 h for ketamine and its metabolites. Dehydronorketamine (DHNK) and (2*R*,6*R*)- hydroxynorketamine (HNK) were still detectable (>4 ng/mL) 1-day post-ketamine infusion (0.5 mg/kg i.v. over 40 min) in patients.^24^ ^,^^55^ Elimination of ketamine primarily occurs via the kidneys, though unchanged ketamine accounts for only a small percentage in the urine.^56^

### NMDA-dependent mechanism

**Figure 1.**
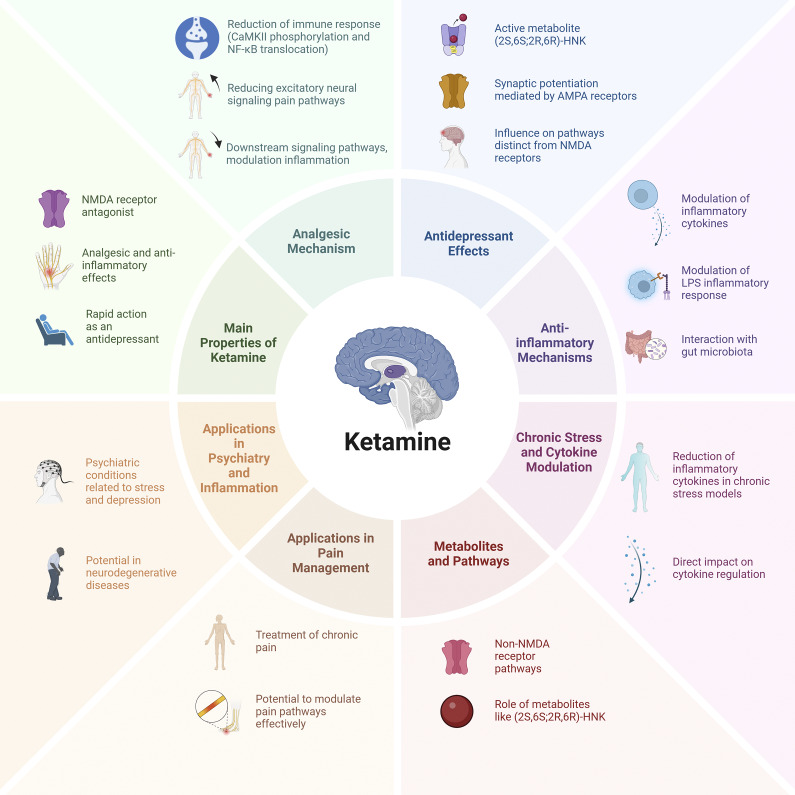
Mechanisms, properties, and applications of ketamine. This figure summarizes the main pharmacological properties, mechanisms of action, and clinical applications of ketamine. The central panel highlights its primary properties, including its role as an NMDA receptor antagonist, its rapid antidepressant effects, and its analgesic and anti-inflammatory properties. The surrounding sections describe key mechanisms such as its analgesic effects (modulation of excitatory neural signaling and inflammatory pathways), antidepressant effects (synaptic potentiation via AMPA receptors and actions of active metabolites such as (2S,6S;2R,6R)-HNK), and anti-inflammatory mechanisms (modulation of cytokines, interaction with gut microbiota, and LPS inflammatory response). Additionally, the figure illustrates ketamine’s role in regulating cytokine levels under chronic stress conditions, its metabolic pathways involving non-NMDA receptor targets, and its applications in psychiatry, inflammation, and pain management. These multifaceted actions contribute to its emerging therapeutic potential beyond anesthesia.

Ketamine primarily exerts its effects through noncompetitive NMDA receptor antagonism, reducing excitatory neurotransmission.^57^ ^,^^58^ NMDA receptor blockade also activates downstream pathways involved in synaptic plasticity and mood regulation, contributing to ketamine’s antidepressant effects.^59^ Its main mechanism involves noncompetitive blocking of the NMDA receptor’s calcium channel, which is key to its anesthetic and analgesic effects in the central nervous system (CNS) and spinal cord. Additionally, ketamine reduces presynaptic glutamate release.^60^ Ketamine, along with other NMDA receptors, binds to the PCP site within the receptor’s channel, reducing the time the channel remains open. This decreases the response amplification to repeated stimuli, which is linked to CNS sensitization. This “wind up” effect is considered an elementary form of CNS desensitization.^61^ Ketamine also binds to a second site on the NMDA receptor’s hydrophobic domain, reducing the frequency of channel openings and acting as an allosteric antagonist. It specifically targets the NR2B subunit of the receptor, which is involved in emotional perception and pain memory.^62^ Ketamine may also inhibit nitric oxide synthase, contributing to its analgesic and anesthetic effects.^47^ Additionally, it can inhibit eukaryotic elongation factor 2 kinase, leading to increased production of BDNF, which enhances synaptic connectivity in the cortex, hippocampus, and nucleus accumbens (NAc). Chronic stress, however, reduces the number of postsynaptic NMDA and AMPA receptors in these regions, weakening synaptic strength.^63^ Ketamine administration quickly counteracts these effects by boosting postsynaptic glutamate activation, increasing neurotrophic signaling, and promoting protein synthesis, all of which help restore synaptic connectivity for prolonged periods^64^ (Figure 1).

With regard to the mechanism of action of NMDA receptors, the main inhibitory neurotransmitter, gamma-aminobutyric acid (GABA), activates ionotropic receptor subtypes GABA_A_ and GABA_C_, as well as the metabotropic receptor subtype GABA_B_ in the brain.^65^ Electrophysiological studies have shown that high concentrations of ketamine enhance GABAergic inhibitory postsynaptic currents in neurons from guinea pig olfactory cortex and rat hippocampal slices. However, at clinically relevant doses in mice, ketamine does not affect GABA uptake. It has also been found that ketamine can directly open GABA_A_ receptors, but only at high concentrations that are unlikely to be reached in clinical settings^24^ ^,^^66^ (Figure 1).

### Opioid receptors

Emerging evidence indicates that μ-opioid receptors (MORs) play a critical role in mediating the antidepressant effects of ketamine. Preclinical studies have demonstrated that MOR antagonists significantly reduce ketamine behavioral efficacy in rodent models, suggesting that endogenous opioid signaling is necessary for its full therapeutic action.^67^ ^,^^68^ Zanos and Gould, as well as Henter et al., have highlighted MOR involvement as part of a broader network of neurobiological processes underlying ketamine’s rapid antidepressant effects.^11^ Clinical data also supports this interaction. Berman et al. observed mood-elevating and analgesic responses in human subjects, which may be partially attributed to MOR activation.^69^ However, this receptor system is also a key component of reward-related neurocircuitry, raising concerns regarding its potential misuse. Niesters et al. emphasize that ketamine’s engagement of MORs could contribute to its reinforcing properties, blurring the line between therapeutic and addictive potential.^70^

In support of this, ketamine has been shown to bind μ-, δ-, and κ-opioid receptors (ORs), although the precise mechanisms underlying its engagement with the endogenous opioid system remain unclear.^24^ In adult male Sprague–Dawley rats, ketamine enhanced β-endorphin release, which activated μ-ORs in the medial prefrontal cortex (mPFC). This interaction appears to be essential for both behavioral and molecular antidepressant responses. This finding aligns with broader evidence linking MOR activity in the mPFC to ketamine’s rapid antidepressant effects.^71^ In the context of analgesia, studies in mice have demonstrated that ketamine’s analgesic effects are blocked by antagonism of μ- and δ-ORs, but not κ-ORs, within the brain. However, in humans, global opioid receptor blockade with naloxone does not abolish ketamine-induced analgesia, suggesting the involvement of indirect opioid system modulation or receptor-subtype-specific interactions.^67^

Given the dual roles of MORs in therapeutic efficacy and abuse liability, future studies should pursue several key avenues. First, disentangling the precise contribution of MORs from that of other neurotransmitter systems (e.g., glutamatergic and GABAergic) is essential to isolate the mechanisms responsible for antidepressant versus reinforcing effects. The use of selective MOR partial agonists or biased ligands may help to identify compounds that retain antidepressant efficacy with reduced abuse potential. Second, neuroimaging studies in humans could provide insight into how MOR activity correlates with clinical outcomes and subjective effects, helping refine patient selection criteria. Third, there is a need to explore interindividual variability, both genetic and epigenetic, in MOR expression or function, which may influence both the therapeutic response and risk of dependence. Finally, longitudinal studies are required to assess whether repeated ketamine exposure leads to neuroadaptive changes in MOR-related pathways, potentially increasing the vulnerability to misuse over time. Clarifying these aspects will be critical for developing safer ketamine-based therapies and informing regulatory frameworks that minimize risks while preserving clinical benefits.

### Monoaminergic system receptors

Although ketamine is believed to primarily target the NMDA receptor, subanesthetic doses also affect monoaminergic neurotransmission in the CNS, including both the serotonergic and dopaminergic systems. Some studies found that ketamine may influence the serotonin transporter and the plasma membrane monoamine transporter by enhancing serotonin signaling via binding to 5-hydroxytryptamine (5HT)1B receptors, contributing to its antidepressant actions.^72^ However, these transporters are not essential for ketamine to exert its effects.^55^

Ketamine has been extensively studied for its potential effects on the dopaminergic system; however, results have been mixed.^64^ Some studies have suggested that ketamine enhances dopaminergic activity by reducing glutamatergic inhibition, leading to increased dopamine release in the nucleus accumbens and elevated firing of ventral tegmental area neurons.^73^ ^,^^74^ This activation may contribute to ketamine’s rapid antidepressant effects by restoring the deficits in dopamine-dependent synaptic plasticity.^75^ However, other evidence suggests that ketamine’s effects on dopamine are indirect. For instance, PET studies found that esketamine reduced D2/3 receptor binding in the striatum^76^ and, despite increasing dopamine release, esketamine did not significantly affect dopamine transporter binding.^77^

Additionally, ketamine does not directly modulate dopamine efflux or bind to D2 receptors.^78^ Although chronic use may alter D1 receptor availability,^79^ its impact on dopamine synthesis and transporter function is minimal. Notably, ketamine may block high-affinity D2 receptors at anesthetic doses,^80^ but this effect appears to be dose-dependent. Overall, the effects of ketamine on the dopaminergic system are primarily mediated through NMDA receptor antagonism and indirect modulation of neurotransmitter networks, including serotonin.^81^ ^,^^82^ Although ketamine can influence dopamine release, its direct interaction with dopamine receptors or transporters is limited, highlighting the need for further investigation.

### Cholinergic receptors

Ketamine directly inhibits cholinergic (both nicotinic and muscarinic) receptors in the prefrontal cortex and acts on cholinergic neurons in the hippocampus and the striatum, which has psychological effects.^83^ As a result, the anticholinesterase agent physostigmine can counteract ketamine’s central anticholinergic effects and reverse its hypnotic actions.^84^

### Neural plasticity

Depression has been linked to synaptic deficits and maladaptive plasticity. The importance of these deficits and whether they cause depression versus are a consequence of depression is not clear. Ketamine appears to have neurotrophic effects, resulting in dendritic spine growth in cortical pyramidal neurons.^85^ Preclinical rodent studies found that these changes occur rapidly,^86^ and it has been speculated that neuroplastic changes may be related to ketamine’s rapid onset of action. However, the evidence is not clear, and much further work is required.

## Ketamine’s therapeutic role

In the past 50 years, ketamine has become a vital anesthetic in humans^87^ and, more recently, its range of uses has increased to include managing chronic pain, treating TRD, and reducing suicidal ideation. The discovery of ketamine’s rapid antidepressant effects has led to a substantial increase in research aimed at understanding its therapeutic benefits. Indeed, ketamine has been one of the most impactful substances in advancing psychiatric disorder research and treatment over the past few decades.^88^

### Anesth Analg

Ketamine stands out among most other sedatives and anesthetics due to its ability to induce a state of dissociative anesthesia by blocking NMDA receptors at high doses, which is a unique quality not commonly found in these types of drugs.^89^ Unlike most other anesthetics that possess sedative or hypnotic properties and primarily act through GABA receptors, ketamine has a different mechanism of action.^90^

Ketamine’s dissociative effects can be characterized by the experience of being conscious while simultaneously detached from sensory perceptions. As the dose increases, the dissociative state intensifies, leading to dream-like states of both open- and closed-eye visuals, as well as significant perturbations in thought and bodily sensation.^91^ Ketamine exerts a substantial influence on the intracortical dynamics of the brain, which can be ascribed to its singular effects. This can be explained by distinct alterations in intracortical dynamics that are evident during ketamine administration.^92^ When administered at high doses, ketamine induces a state of deep dissociation that is accompanied by amnesia and a loss of consciousness. This effect occurs because ketamine affects NMDA receptors, which are critical components of excitatory neurotransmission, long-term potentiation, and memory formation.^93^ Ketamine-induced analgesia and ketamine-induced dissociation are both independently regulated by ketamine and do not exhibit a robust intrinsic connection.^94^

In addition, research has demonstrated that ketamine exhibits distinct patterns of neural activity compared to other anesthetics. The effects of ketamine on the electrophysiological activity of the brain can be quantified using electroencephalography (EEG). In humans, the disappearance of behavioral responsiveness coincides with the emergence of EEG slow-wave activity.^95^ The results of high-density EEG studies conducted on human volunteers demonstrate that power in the theta, gamma, and delta frequency bands increased in both frontal and posterior channel clusters during ketamine anesthesia.^96^ Posterior alpha power was also found to be decreased under both anesthetic and subanesthetic dose conditions.^96^

Due to its non-GABAergic mechanism of action, ketamine is a reliable and effective option for emergency anesthesia in a prehospital setting. Its wide dosing range makes it suitable for various situations, and its sympathomimetic effect helps maintain cardiovascular stability and respiratory function. Ketamine also provides a level of analgesia comparable to that of morphine, making it an excellent choice for pain management in emergency situations.^5^ ^,^^97^ During dissociative anesthesia, fundamental reflexes stay intact, pulmonary compliance increases, and airway resistance and bronchospasm decrease, benefiting patients with airway diseases.^98^ For the induction of anesthesia, racemic ketamine is typically given intravenously at a dose of 1–2 mg/kg (Figure 1), resulting in dissociative anesthesia within a period of 1–2 min. This administration is typically carried out as a bolus.^5^

As noted above, ketamine can be effectively administered through various routes, including intravenous, oral, sublingual, intranasal, intramuscular, intraosseous, rectal, inhaled, and subcutaneous. Intravenous administration is the most effective route in terms of bioavailability and onset time.^5^ ^,^^99^ Currently, ketamine is employed in emergency departments for anesthesia and procedural sedation in diverse patient populations ranging from minors to adults. In this context, ketamine’s extensive dosing range and analgesic properties, in addition to the possibility of administering intramuscular doses, have proven particularly advantageous and enable its administration in field conditions where no anesthesiologist or monitoring equipment is accessible.^100^ ^,^^101^ However, ketamine is not typically used as the primary agent for general anesthesia in hospitals because of its psychotomimetic effects and potential to cause emergence phenomena. Emergence phenomena, which can manifest as profound confusion or hyperexcitation upon emerging from the dissociative state, affect up to 20% of the patients.^97^

Administering 0.5 mg/kg of ketamine upon induction followed by 10 μg/kg/h until wound closure decreased perioperative opioid requirements in opiate-dependent patients with chronic back pain undergoing back surgery.^102^ Similarly, perioperative IV ketamine administration likely reduces postoperative pain and analgesic use, with consistent results across surgeries, dosing, and pain levels, with minimal CNS adverse effects.^103^ A systematic review published in 2017 showed that intravenous ketamine administration significantly reduced postoperative hysterectomy pain.^104^

Another systematic review showed that perioperative intravenous ketamine administration can reduce postoperative analgesic consumption and pain intensity.^105^ The results were consistent for different types of operations and for the timing of ketamine administration, with larger and smaller studies and higher and lower pain intensity. CNS adverse events differed slightly between the ketamine and control groups. Perioperative intravenous ketamine can also reduce postoperative nausea and vomiting to a small but clinically relevant extent.^106^ Ketamine has also been shown to effectively reduce opioid requirements and postoperative nausea and vomiting in patients undergoing bariatric surgery, particularly in those with high pain levels or opioid-related vulnerabilities.^107^ It may also attenuate postoperative hyperalgesia, although further research is necessary to confirm this finding.^104^ ^,^^108^

### Pediatric use

Administering a 0.5 mg/kg dose of ketamine as an adjunct to local anesthetics for caudal block is both safe and effective in managing postoperative pain in children.^109^ Furthermore, a meta-analysis found that pediatric patients receiving caudal ketamine experienced reduced postoperative pain and a lower need for non-opioid analgesics.^110^ Additionally, a comprehensive systematic review of randomized controlled trials that incorporated ketamine into pediatric caudal anesthesia found that it extended the duration of pain relief while causing minimal side effects, in contrast to the use of local anesthetic alone.^111^

In pediatric cleft palate surgery, the use of bupivacaine or ketamine at the surgical site can provide effective pain relief with minimal side effects. While both options are beneficial, ketamine appeared to outperform bupivacaine in reducing the need for additional analgesics, promoting better sleep and allowing for earlier feeding.^112^ Furthermore, a study found that administering ketamine, either subcutaneously or intravenously, at the end of tonsillectomy surgery safely helped manage post-operative pain without significantly increasing the risk of complications.^113^ In the context of children with burns, the application of oral midazolam and ketamine offered superior analgesic effects than midazolam, acetaminophen, or codeine for painful procedures.^114^ A recent investigation that explored the use of adjunctive ketamine and morphine to alleviate postoperative pain and reduce opioid usage in adolescent patients undergoing spinal surgery found decreases in morphine consumption, pain scores, and nausea, suggesting that ketamine had a positive impact on postoperative outcomes.^115^

### Clinical implications of ketamine anesthesia requiring further study

Although the use of ketamine has shown promising results in many studies, it is important to note that not all trials have reported positive outcomes. For instance, one study found that the administration of low-dose ketamine (10 mg i.v.) combined with spinal bupivacaine, fentanyl, morphine, and i.v. ketorolac after cesarean section provided no additional postoperative analgesic benefit.^116^ Another study found that adding 0.25–1 mg/kg ketamine to anesthesia induction for cesarean section did not improve postoperative analgesia.^117^ Similarly, a combination of ketamine (5 mg/kg/min) and remifentanil for cholecystectomy did not improve postoperative pain severity, time to first analgesic supplementation, or total morphine requirement within 24 h.^118^ In addition, adding ketamine (5 μg/kg/min) did not prevent or reduce remifentanil-induced postoperative hyperalgesia.^119^ Also, while propofol–ketamine versus propofol–alfentanil for dilatation and curettage showed comparable results, the ketamine group required more time before orientation returned.^120^ A different study found that propofol/alfentanil and propofol/ketamine combinations provided adequate levels of hypnosis and pain relief during upper gastrointestinal endoscopy in severely obese individuals, but the propofol/ketamine mixture led to significantly higher consumption of propofol.^121^

The findings indicate that ketamine’s efficacy in improving *Anesth Analg* may be limited, necessitating additional research to determine its appropriate use. Given the considerable clinical variability, it is too early to recommend a standardized ketamine protocol. Further investigation is needed to address questions about optimal dosing, treatment duration, and patient-centered outcomes, including long-term effects. These areas require further exploration in future research to fully comprehend the role and effectiveness of ketamine in anesthesia and acute pain management.^105^

### Chronic pain management

Chronic pain is typically caused by central sensitization or neuropathic processes that lead to hyperalgesia or allodynia.^122^ ^,^^123^ Ketamine’s effects on NMDA receptors are crucial for its effectiveness in the management of chronic pain.^124^ Ketamine has been used as a therapeutic option for managing persistent pain disorders, particularly those with a neuropathic nature and pain arising from cancer,^125^ ^,^^126^ However, the results of ketamine research on chronic pain are promising but inconsistent, requiring additional rigorous studies to determine optimal use and long-term consequences. Although ketamine causes few deleterious effects, the interdependence between dose, efficacy, and safety profile warrants precise delineation.^127^

Low-dose ketamine effectively relieves neuropathic pain by inhibiting NMDA receptors; modulating serotonin, dopamine, and norepinephrine reuptake; and enhancing descending inhibition and central anti-inflammatory effects.^128^ One study found that postoperative ketamine at 0.2 mg/kg/h combined with opioids significantly reduced the average pain scores in surgical patients on chronic opioids. However, it did not affect the lowest or highest reported pain levels or postoperative opioid consumption, indicating a limited overall pain management benefit.^129^ In contrast, a study of opioid-tolerant patients undergoing spinal fusion surgery who received low-dose ketamine infusion for the first 24 h found that these patients required fewer opioids than those who did not receive the infusion. However, this effect was not observed in opioid-naïve patients.^130^ A meta-analysis on the efficacy and safety of perioperative ketamine for the prevention of chronic postsurgical pain showed low-certainty evidence, suggesting that perioperative ketamine does not affect chronic postsurgical pain in adults. Similarly, compared to placebo, ketamine may reduce the occurrence of chronic postsurgical neuropathic pain after three months^131^; however, optimal dosing, treatment duration, and impact on patient-related outcomes remained unclear, highlighting the need for additional research.

Subanesthetic ketamine infusions offer a promising treatment option for chronic pain of both neuropathic and nociceptive origin that is refractory to conventional treatments. Studies have demonstrated that these infusions can modestly improve pain outcomes while also presenting common yet mild side effects that can be effectively managed through pharmacological interventions.^132^ A recent systematic review and meta-analysis conducted to assess the effectiveness and safety of ketamine as a treatment for cancer pain in adult patients found that it holds promise for reducing the severity of cancer pain, decreasing opioid use, and possibly ameliorating depressive symptoms.^133^ Nevertheless, additional robust clinical trials with larger sample sizes are necessary to verify these findings and to establish the optimal dosage and administration route for ketamine in cancer pain management. Ketamine has also shown promise as a potential treatment for various persistent pain disorders, including neuropathic pain, fibromyalgia, CRPS, phantom limb pain, cancer pain, and post-thoracotomy pain syndrome. Studies have revealed its efficacy in reducing pain symptoms, enhancing patient satisfaction, and improving overall quality of life in these conditions.^134^

### Psychiatric use

As a rapid-acting antidepressant, ketamine represents a paradigm shift in neuropsychiatric care, providing swift relief from depressive symptoms within hours. Robust clinical evidence supports the efficacy of subanesthetic doses of ketamine and its enantiomer, esketamine ((*S*)-ketamine). The (*S*)- and (*R*)-enantiomers of ketamine exhibit distinct pharmacological properties that contribute to their therapeutic potential in a broad range of neuropsychiatric disorders, including various forms of depression, anxiety, substance use, and eating disorders. Additionally, ketamine has demonstrated rapid efficacy in alleviating core symptoms of depression, such as anxiety, anhedonia, and suicidal ideation.^35^ Ketamine’s ability to effectively reduce the symptoms of major depressive disorder and bipolar disorder has been extensively reviewed.^135^ ^–^^137^ And, indeed, a recent study involving 403 patients with nonpsychotic TRD, randomized across five clinical sites, with 200 patients assigned to the ketamine group and 203 to the ECT group, found that ketamine was noninferior to ECT, the current gold standard for TRD without psychosis.^138^

### Treatment-resistant depression

As previously mentioned, ketamine differs from traditional antidepressants in that it targets NMDA receptors in the glutamatergic system, enhances synaptic plasticity, and rapidly alleviates depression through unique neurochemical mechanisms.^139^ ^–^^141^ Although both ketamine and esketamine hold promise for TRD, their adverse effects, patient selection, and monitoring must be carefully considered. For esketamine, the most common route of administration is intranasal, typically in combination with a newly initiated antidepressant; for ketamine, intravenous administration of racemic ketamine as monotherapy or adjunctively with preexisting psychotropic treatments is the most common way of delivering ketamine, and the most frequently explored. Numerous short-term randomized controlled trials have consistently demonstrated the rapid and significant effectiveness of both formulations and modes of administration in adults with TRD. The efficacy of a single dose of ketamine lasts 3–7 days, while repeated intravenous racemic ketamine is effective for up to 2–3 weeks; most patients relapse within 1 month (median, 18 days), necessitating repeated administration.^142^

Esketamine’s recommended dose for intranasal use is 56 mg on the first day, with the possibility of increasing to 56–84 mg twice weekly for the initial 4 weeks. For the subsequent 4 weeks, the dosage is adjusted to 56–84 mg once weekly, followed by every 1–2 weeks thereafter. For treating depressive symptoms in adults with major depressive disorder and acute suicidal ideation or behavior in the US, the recommended dose is 84 mg twice per week for 4 weeks. After week 4, it is important to evaluate the treatment’s therapeutic benefits, and if minimal response is observed, discontinuation may be recommended.^143^ Despite a systematic review and meta-analysis revealing that intravenous ketamine was more effective than intranasal esketamine in treating depression, it is important to note that these findings should not be interpreted as definitive or prescriptive.^144^ The adverse effects most often experienced during esketamine treatment included nausea, dissociation, dizziness, vertigo, numbness, sedation, and a tingling sensation.^145^ Safety concerns include risks such as bladder damage and, in rare cases, suicidal behavior.^146^ A recent pharmacovigilance analysis by Jiang et al. (2023), based on the FDA Adverse Event Reporting System (FAERS), highlighted several potential concerns associated with intranasal esketamine, including reports of dissociation, sedation, suicidal ideation, and suspected dependence. The study emphasizes the importance of ongoing surveillance regarding esketamine’s long-term safety, particularly with respect to addiction potential and sustained antidepressant efficacy.^147^ However, long-term data extending up to 7 years indicate low rates of suicidal behavior, providing important insight into esketamine’s long-term safety profile.^148^

Nevertheless, further research is needed to fully understand the long-term consequences, comparative effectiveness, and strategies to maximize the effectiveness of both ketamine and esketamine. Additional studies are needed to explore the possible benefits of these drugs in other depressive disorders and investigate their combination with psychosocial treatments and other rapid-acting antidepressant medications.^142^

### Impact of ketamine on suicide

Both ketamine and esketamine have been shown to rapidly reduce suicidal ideation,^149^ ^,^^150^ with benefits observed after both single and repeated doses.^151^ Notably, ketamine’s antisuicidal effects were found to occur within 2 h,^152^ endure for up to 72 h and last 7 days or more^153^ ^,^^154^; these effects were sustained through maintenance doses, indicating the potential for short-term management of suicidal thoughts, with further research needed for long-term benefits.^152^ Furthermore, these effects appeared to occur independently of its antidepressant effects.^155^ ^,^^156^ Another randomized, double-blind clinical trial that looked at both ketamine and esketamine found that both drugs were effective in rapidly reducing suicidal ideation in individuals with TRD at 24 h and up to 7 days post-infusion; no significant differences in efficacy were observed between ketamine and esketamine.^157^

Overall, compelling evidence supports a favorable short-term risk–benefit profile for intravenous racemic ketamine, while the risk–benefit balance of intranasal esketamine remains under evaluation for safety and long-term efficacy, despite its proven effectiveness in reducing suicidal thoughts. However, esketamine offers a potentially more convenient intranasal option, and the growing body of research supporting its efficacy and safety profile led to FDA approval for the treatment of major depressive disorder with suicidal ideation or behavior.^158^ ^–^^160^ The available data does not provide information on the impact of maintenance of esketamine or ketamine treatments with suicidality as the primary measure.^142^ In this context, the integration of ketamine into a comprehensive multimodal treatment strategy for patients with suicidal tendencies requires careful clinical application and continuous pharmacovigilance.^161^

In addition to its therapeutic effects, ketamine has shown promise as a prophylactic agent in preclinical and early clinical studies. Research indicates that a single administration of ketamine prior to a stressful event may reduce the subsequent development of depression-like or PTSD-related behaviors, possibly by enhancing stress resilience mechanisms.^162^ ^–^^164^ These findings open new avenues for preventive mental health strategies, particularly in high-risk populations.

## Tolerability and safety

The adverse events that may occur during treatment with ketamine for anesthetic/analgesic and psychiatric uses can be classified into several categories, including psychiatric, neurologic/cognitive, hemodynamic, genitourinary, and abuse liabilities.^165^ ^,^^166^ With regard to ketamine versus esketamine for the treatment of depression, side effects are typically identical in terms of both the percentage and severity of events.^1^ Side effects include headaches, dizziness, dissociation, elevated blood pressure, blurred vision, and anxiety; these effects usually occur immediately after treatment and resolve quickly.^167^ The variation in the occurrence and intensity of adverse events is influenced by factors such as differences in ketamine formulation, administration route, patient demographics, coadministered medications, and study design features. However, intravenous ketamine-related adverse events have not always been consistently documented, and the available data may be biased due to limited information on long-term exposure to ketamine. In contrast, safety and tolerability information for esketamine in TRD is comprehensive, covering both short- and long-term exposures.^137^

### Adverse psychiatric effects

*Dissociation:* The psychotomimetic effects of ketamine during anesthetic use, including auditory hallucinations, paranoid ideas, anxiety, inability to control thoughts, derealization, visual hallucinations, and heightened sensitivity to sound and color, are dose-dependent and may vary according to the administered dose.^70^ With subanesthetic ketamine infusions, the risk of psychotomimetic effects occurs in approximately 1 out of every 21 patients. The incidence rises with faster infusion rates but declines rapidly once the infusion is stopped.^168^ Another study found that patients receiving ketamine for pain who had a history of depression had a lower incidence of ketamine-related adverse effects than those with no history of depression (10.3% versus 37.3%).^169^

With regard to ketamine use in TRD populations, psychotomimetic effects, which are also dose-dependent, include dissociation, perceptual disturbances, odd sensations, derealization, hallucinations, feelings of strangeness, and depersonalization.^166^ Typically, dissociation peaks within 40 min of administration and tends to subside within the time frame of 1 to 2 h. Approximately 72% of studies using intravenous racemic ketamine for TRD reported dissociation, which is significantly higher than the 36% reported in studies using non-intravenous racemic ketamine.^166^ This difference is likely due to differences in plasma levels rather than the route of administration.^166^ However, the proportion of individuals with TRD who reported dissociation diminished with each subsequent administration.^166^ Interestingly, dissociation is neither necessary nor sufficient to elicit an antidepressant response.^170^ ^,^^171^

It should be noted that the Clinician-Administered Dissociative States Scale (CADSS) is the most widely used tool for gauging the intensity of dissociation in TRD cases.^142^ ^,^^172^ There is agreement that the CADSS, which has been adapted for use as a safety measure for ketamine, does not adequately assess the full range of psychotomimetic experiences associated with ketamine and likely underestimates the occurrence of dissociation.^173^ Despite its limitations, as it lacks validation against other safety measures, the KSET demonstrates face and content validity, and is recommended for monitoring acute and long-term ketamine side effects.^172^

*Induction of psychosis:* Ketamine can produce symptoms similar to psychosis, including hallucinations, delusions, and cognitive impairments, resembling those observed in schizophrenia when used in healthy volunteers.^174^ ^,^^175^ This may hold particular significance, especially for those who have preexisting vulnerabilities. According to previous reports, individuals with a history of psychosis are more likely to experience dissociation when administered ketamine.^176^ However, these individuals are not prone to developing psychosis because of ketamine use. Despite their increased likelihood of dissociation, the duration of this experience did not extend beyond 40 min.^176^ However, ketamine can cause psychotic symptoms in individuals with schizophrenia and depressive disorders.^177^ ^,^^178^ Nevertheless, esketamine showed robust antisuicidal and antidepressant effects in a schizophrenic patient with severe depression, without causing psychotic symptoms, suggesting its broader therapeutic potential.^179^

*Neurologic/cognitive:* Ketamine’s neurological and cognitive effects range from psychedelic experiences, such as auditory hallucinations, to disorientation in time and space, along with physical symptoms such as dizziness and nausea. Although memory loss in new users typically resolves on its own, prolonged, and excessive ketamine use for ≥12 months is known to have a detrimental impact on cognitive abilities and exacerbate psychological issues, highlighting the importance of user education and healthcare interventions,^180^ particularly in the context of low-dose ketamine use on memory in chronic pain management.^70^ Nevertheless, in adults with TRD, there have been no consistent or repeated reports of impaired cognitive functioning in those treated with racemic ketamine or intranasal esketamine.^181^ ^,^^182^ Long-term data on esketamine indicate that cognitive performance generally remains stable or improves postbaseline, with a low incidence of significant cognitive impairment in patients with TRD.^148^

*Hemodynamic:* Ketamine and esketamine have dual actions on the cardiovascular system, directly causing a negative inotropic effect while also indirectly stimulating it.^183^ ^,^^184^ In anesthetic and pain management, activation of the sympathetic system leads to the release of catecholamines, inhibition of the vagus nerve, and release of norepinephrine from the sympathetic ganglia. This results in myocardial depression within minutes to hours of high-dose ketamine infusion or repeated ketamine doses. Cardiovascular stimulation, characterized by tachycardia, systemic and pulmonary hypertension, and increased cardiac output and myocardial oxygen consumption, occurs after low-dose ketamine infusion.^183^ ^,^^185^ According to the data provided, it is necessary to closely monitor patients who are being treated with low-dose ketamine. However, whether clonidine or beta-adrenoceptor blockade can improve hemodynamics following ketamine treatment has not been investigated yet, despite it being plausible.^70^

The adverse hemodynamic event most frequently observed with ketamine use in depression is an elevation in heart rate and blood pressure, which may be accompanied by palpitations, arrhythmias, chest pain, and hypotension.^186^ Roughly 10–50% of patients experience increased systolic and diastolic blood pressure after treatment, which typically occurs within 20–50 min and resolves within 2–4 h. Notably, one study found that administering ketamine for the treatment of depression over a period of 40 min at a dose of 0.5 mg/kg resulted in only small, well-tolerated, and clinically insignificant changes in blood pressure.^187^ Nevertheless, 20–30% of individuals receiving ketamine (usually intravenous for TRD) may have blood pressures above 180/100 mmHg and/or heart rates of ≥110 beats per minute.^166^ ^,^^186^ One study found that approximately 20% of individuals receiving ketamine for TRD in a community-based clinic may need pharmacological treatment for intravenous ketamine-induced hypertension, depending on the clinic-level protocols in place.^188^ In contrast, the rate of hypertension in the esketamine development program for TRD was relatively low, with only 2.1% of patients requiring antihypertensive treatment, compared to 1.2% in the placebo group.^189^ However, it is important to note that these participants were enrolled in clinical research settings, where the inclusion criteria likely excluded individuals with poorly controlled blood pressure, which may have influenced the observed rates.^189^ Although hemodynamic changes are generally asymptomatic, they may not subside with subsequent ketamine administration.

*Genitourinary:* Ketamine use, especially prolonged or heavy use, may result in severe genitourinary effects, including symptoms of ketamine-induced ulcerative cystitis. These symptoms include increased urgency and frequency of urination, dysuria, urge incontinence, and hematuria.^70^ Chronic ketamine abuse can alter bladder function, leading to severe urological issues in users who abuse the drug at least three times a week for 2 years or more.^190^ Disruption of the urine-bladder epithelial interface, bladder neuromuscular junction destruction, nitric oxide synthase-mediated inflammation, and immunoglobulin E-mediated inflammation characterize the pathology underlying lower urinary tract symptoms.^191^ Bladder cystoscopy revealed signs of inflammation of the bladder wall, whereas further tests revealed instances of thickened ureters, stenosis, vesicoureteral reflux, and hydronephrosis.^192^ ^,^^193^ Approximately 20% to 40% of individuals who use ketamine recreationally experience lower urinary tract symptoms.^194^ ^,^^195^ Long-term ketamine use may result in persistent symptoms in approximately 5% of individuals, even after drug cessation.^192^ ^,^^196^

In this context, it should be noted that prolonged ketamine exposure, often necessary for individuals with depression, may result in adverse effects, as demonstrated by the dose–response relationship between ketamine exposure and the likelihood of experiencing lower urinary tract symptoms. The fact that some studies involving intranasal esketamine have not reported a significant number of individuals experiencing genitourinary issues is a positive development.^148^ ^,^^191^ Currently, there is no universally accepted treatment method for ketamine-related kidney, ureter, bladder, and related pathologies other than the temporary cessation of ketamine administration.^197^

*Abuse liability:* Ketamine’s popularity as a recreational drug is largely attributed to its psychedelic side effects—the same limitations that restrict its clinical use. However, unlike other drugs that result in physical dependence and subsequent withdrawal symptoms, ketamine does not produce any observable physical withdrawal state upon discontinuation of long-term abuse.^185^ Ketamine is often used by individuals who ingest, snort, or inject it at relatively high doses, which can result in an experience that typically lasts no more than 2 h. When the dissociative effects of ketamine are severe, the individual may experience a phenomenon known as the K-hole, which is characterized by symptoms that resemble schizophrenia, such as perceived perceptions that are completely separate from reality (e.g., near-death experiences). At lower doses, the drug can induce a state of mild dissociation, accompanied by vivid hallucinations and distortion of time and space, such as the sensation of melting into one’s surroundings or experiencing an out-of-body sensation.^185^ ^,^^198^

Ketamine is categorized as a Schedule III substance in the United States because of its potential misuse and, in the UK, ketamine is a Class C drug. Racemic ketamine, administered intravenously for TRD at doses of 0.4–0.8 mg/kg, has been found to increase preference for the drug, leading to concerns about drug misuse and sensitivity to other misuse drugs in healthy individuals.^6^ ^,^^180^ ^,^^199^ ^,^^200^ Both intravenous racemic ketamine and esketamine can lead to an increase in drug preference among recreational polydrug users.^201^ However, the likelihood of recreational ketamine abuse has not been shown to increase with the use of esketamine.^148^

A previous study indicated that chronic ketamine users receiving lamotrigine, a glutamate release inhibitor, experienced a significant reduction in the frequency and daily dose of ketamine.^202^ Although ketamine is a medicinal substance with a generally safe pharmacological profile, its abuse has severe consequences for both individuals and society.^203^

Conclusions: Ketamine has undergone a remarkable transformation from its initial use as an anesthetic to its current role as a novel therapeutic agent in psychiatry and pain management. Its rapid and robust antidepressant effects, particularly in TRD, have revolutionized the field of mental health, offering hope to patients who do not respond to conventional treatments. Additionally, its analgesic, anti-inflammatory, and neuroprotective properties highlight its broader clinical utility beyond anesthesia. Despite its significant therapeutic potential, ketamine presents challenges, including its dissociative and hallucinogenic effects, which contribute to its misuse and regulatory restrictions. Prolonged recreational use has been associated with serious health complications, necessitating careful monitoring and risk mitigation strategies. The balance between medical benefits and the potential for abuse remains a critical concern for healthcare providers and policymakers.

Ongoing research continues to deepen our understanding of ketamine’s mechanisms of action, long-term safety profile, and emerging therapeutic applications, including its use in bipolar depression, post-traumatic stress disorder, and substance use disorders. Future studies should aim to optimize dosing strategies, develop alternative delivery methods, and identify predictive biomarkers of treatment responses. As scientific knowledge advances, the clinical integration of ketamine must be guided by rigorous evidence with the goal of maximizing therapeutic benefits while minimizing associated risks.

The evolving understanding of the mechanisms, therapeutic potential, and risk profile of ketamine highlights the critical importance of sustained investigation. Further research into its antidepressant effects, prophylactic potential, abuse liability, and long-term safety is essential not only for advancing scientific knowledge but also for improving clinical care. Continued generation of robust evidence will be the key to informing best practices, shaping regulatory decisions, and ensuring safe and effective use across diverse patient populations.
